# Smart Mesoporous Silica Nanoparticles for Protein Delivery

**DOI:** 10.3390/nano9040511

**Published:** 2019-04-02

**Authors:** Hai-Jun Liu, Peisheng Xu

**Affiliations:** Department of Discovery and Biomedical Sciences, College of Pharmacy, University of South Carolina, 715 Sumter, Columbia, SC 29208, USA; HAIJUN@mailbox.sc.edu

**Keywords:** mesoporous silica nanoparticle, protein delivery, controlled release, stimulus responsive MSN

## Abstract

Mesoporous silica nanoparticles (MSN) have attracted a lot of attention during the past decade which is attributable to their versatile and high loading capacity, easy surface functionalization, excellent biocompatibility, and great physicochemical and thermal stability. In this review, we discuss the factors affecting the loading of protein into MSN and general strategies for targeted delivery and controlled release of proteins with MSN. Additionally, we also give an outlook for the remaining challenges in the clinical translation of protein-loaded MSNs.

## 1. Introduction

Recombinant human insulin (humulin), the first recombinant protein therapeutic was developed and launched by Herbert Boyer, and Eli Lilly opened the Pandora’s box in pharmaceutical proteins 30 years ago [1]. Thus far, there are more than 130 pharmaceutical proteins have been approved by the US Food and Drug Administration (FDA) [2], and large quantities of candidates are being tested in preclinical and clinical development, providing effective treatments and diagnostics for almost every field of diseases and disorders, such as cancer, immunological diseases, infectious disease, and neurogenic disease. [3]. Not only do the primary amino acid sequences of pharmaceutical proteins endow their specific therapeutics but also their sophisticated three-dimensional or spatial structures. Two sides of a coin, this delicate spatial structure of proteins is also the root of a great challenge which hampers the use of protein therapeutics, referring to their susceptibility and frangibility to physical and chemical degradation, as well as physical unfolding, misfolding, and aggregation, resulting in decline or loss of biological activity, immunogenicity and relatively short half-life [4,5,6]. When protein therapeutics are orally and transdermally administrated, limited bioavailability even ineffectiveness is another enormous challenge owing to poor membrane permeability, large molecular size, generally negative charge, as well as of hydrophilicity property [7,8]. In the past decades, extensive efforts have been involved in the design and construction of a versatile therapeutic protein delivery system to overcome the above dilemma and improve their pharmacokinetic and pharmacodynamic properties [5], such as chemical modification [9,10], or encapsulation with polymeric hydrogels, lipid-based protein formulations [11], nanoparticles [12], and microspheres [13,14]. Due to their unique chemical, physical and biological properties, nanoparticles such as liposomes, polymer micelles, polymersomes, inorganic nanoparticles (silica nanoparticles, magnetic nanoparticles, and gold nanoparticles), etc. have gained extensive attention in the field of as a vector for the delivery of protein [15]. Nanoparticles, whose size ranges between 1 and 1000 nm, comprising of natural materials, synthesized materials or their hybrids can entrap or encapsulate, adsorb, or attach therapeutic drugs or biologically agents, thereby the pharmacokinetic behavior of protein therapeutics could be partially substituted or determined by the property of nanoparticles, including composition, size, surface potential, and ligand decoration and so on.

Generally speaking, all of these kinds of nanoparticles could achieve relatively improved protein therapeutic benefits in some degree, including: (a) protect protein from premature degradation, (b) prolong protein circulation time, (c) mask their epitope and subsequent immunogenicity, (d) realize their targeted delivery and controlled release, (e) enhance therapeutic efficacy, whereas reducing side effects. However, premature protein leakage, degradation, and limited release deriving from the soft property of liposomes, degradation of polymer matrixes, and chemical modification, respectively, are major challenges that these nanoparticles have to address when they serve as protein drug-delivery vehicles.

Due to its ease of synthesis, facile surface multi-functionalization, preeminent biocompatibility as well as excellent physicochemical and thermal stability, mesoporous silica nanoparticles (MSN) have gained great attention as excellent delivery vehicles [16,17,18,19,20] for drugs, genetic materials, and protein biomacromolecules since the first MCM-41 type MSN was synthesized by Mobil in 1992 [21]. Additionally, MSN can also realize controlled release and targeted delivery of cargoes by optimizing their size, shape and surface modification [19,22,23]. Many reviews have exhaustively summarized the synthesis of MSN and applications in drug delivery, whereas fewer involved in protein delivery. In this review, we will give an overview of the application of MSN in therapeutic protein delivery, discuss factors affect the loading of protein to MSN, and also demonstrate general strategies for targeted delivery and controlled release of proteins with MSN. Additionally, we will also look at MSN in co-delivery of protein and other drugs and clinic translations for protein.

## 2. Mesoporous Silica Nanoparticles (MSN) as Carriers for Protein Drugs

Since the first discovery of MCM-41 type MSN in 1992 [21] and the first report of MSN as a drug-delivery vehicle in 2001 [24], it has gone through booming development in its synthesis and application in biomedical field. Mesoporous silica nanoparticles, frequently spherical morphology, are commonly synthesized by condensation of organosilane precursors around organic micelles via a base-catalyzed sol-gel process. After removing or etching the template micelle, ordered arrays of hexagonal or honeycomb-like mesoporous channels would appear in the silicon oxide matrix. Within the MSN family, SBA-15 and MCM-41 are the two types of most commonly used MSNs as a protein delivery system. By altering the synthetic condition, such as pH [25], the ratio of silica precursor to surfactant [26], pore expanding agent incorporation [27,28], and hybrid of a template or solution [29,30], its size, pore diameter, and morphology can be tuned. Furthermore, the facile functionalization of MSN with organic or inorganic moieties, typically carried out by one-pot synthesis (co-condensation) and/or post-synthesis (grafting), makes it easy to realize designed loading and controlled release of specific cargoes. For example, Lin and co-worker synthesized an MCM-41 MSN with a large pore size (5.4 nm), and demonstrated that it could not only delivery cytochrome c, a cell membrane impermeable protein into Hela cells, but also could release it intracellularly [31]. Moreover, the released cytochrome c still reserved its catalytic activity. Generally speaking, there are several superiorities by using MSN as a protein delivery system: i) High protein-loading capacity can be achieved with the large pore volumes (>1 cm^3^/g) of MSNs. ii) The chemically and mechanically stable inorganic oxide framework of MSNs shelters the cargo protein from exposure to harmful species. iii) It has been previously proved that MSNs are capable of escaping endo-lysosomal entrapment due to the pH buffering properties [31,32,33]. The following Table 1 gives a summary of the application of MSN in protein delivery.

## 3. The Factors that Affect Protein Loaded into MSN

### 3.1. Pore Size

**Figure 1:**
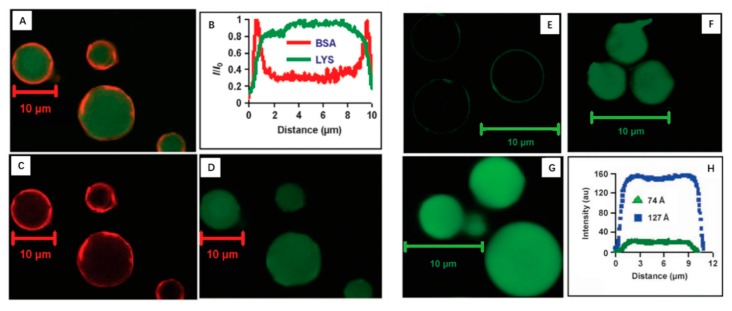
The pore size-selective adsorption and separation of proteins on spherical SBA-15 mesoporous silica nanoparticles (MSN) at pH7.1 (**A**–**D**). (**A**) Merge confocal scanning laser microscopy (CLSM) image of protein adsorbed in SBA-15 (127 Å) (red, Texas Red labeled bovine serum albumin (BSA); green, Alexa Fluor 488 labeled lysozyme (LYS)). (**B**) Normalized protein density along with distance. (**C**) BSA image. (**D**) LYS image. The LYS adsorption in SBA-15 MSN with different pore sizes (**E**–**H**). (**E**) SBA-15 MSN (28 Å). (**F**) SBA-15 MSN (74 Å). (**G**) SBA-15 MSN (127 Å). (**H**) LYS intensity in 74 Å and 127 Å SBA-15 [55].

Pore size is one of the most important parameters of MSN for loading guest molecules, especially biomacromolecules, such as proteins. The pore opening is regarded as a size-selected adsorption characteristic of drugs. When a protein’s size is larger than the pore diameter of a MSN, excepting some protein adsorption on the surface of MSN, the frame work of the MSN is void due to its inaccessibility. Thus, the huge volume and large internal surface area are not sufficiently utilized. Katiyar and Pinto reported the first visualization of a protein–protein separation using SBA-15 materials based on the size difference between the pore and proteins (lysozyme (LYS) and bovine serum albumin (BSA)) [55]. BSA and proteins lysozyme were labeled with Texas Red and LYS Alexa Fluor 488, respectively, and then co-incubated with different MSNs. By using confocal scanning laser microscopy (CLSM), it clearly showed that the smaller protein lysozyme was adsorbed into the pores of SBA-15, whereas the larger BSA was restricted outside the surface of SBA-15, not inside cavity of the pores (Figure 1), giving a visual representation of the influence of a pore on protein loading. Recently, Qiu and co-workers further synthesized three kinds of MSNs with ultra large pores (20, 33 and 40 nm respectively) and interconnected channel structures by using Brij-56 and Brij-97 as templates and ethyl acetate (EA) and dimethyl o-phthalate (DOP) as additives [56]. These MSNs also showed effective performance in size-selective adsorption of 50–55, 60–65 and 70–100 kDa proteins, respectively, demonstrating great potential in protein load and separation. Therefore, it becomes absolutely necessary to optimize the pore size of MSN depending on the size of the protein. Generally, the pore size is mainly determined by the type and property of surfactant templates and pore expanding agent, and the reaction condition such as temperature also has great influence on pore size.

By using an environmentally friendly pore-forming agent tannic acid (TA) as a non-surfactant template, Gao and Zharov demonstrated a one-pot synthesis of a novel large interconnected pore MSN(TA-MSN) [57]. This kind of TA-MSNs possessed a uniform diameter of 200 nm, large pores (6–13 nm) morphology. Moreover, TA-MSN showed a much higher protein loading capacity of 77.1 mg/g for lysozyme, 396.5 mg/g for bovine hemoglobin (BHb), 130.0 mg/g for bovine serum albumin, 421 mg/g for mitochondrial malate dehydrogenase (m-MDH), demonstrated their great potential for biomedical and catalysis applications.

A variety of additives have been employed as pore size expanding agents, such as *N*,*N*-dimethylhexadecylamine (DMHA) [58], trimethylbenzene (TMB) [58,59], aromatic hydrocarbons [60], axiliary alkyl surfactant [61], long-chain alkanes [62,63]. Lin et al. first synthesized and characterized an MCM-41-type mesoporous silica nanoparticle (MSN) material with a large average pore diameter (5.4 nm) by using mesitylene as a pore expanding agent, and employed it as a transmembrane delivery vehicle for proteins [31]. They demonstrated that the MSN could host a cell-membrane-impermeable protein cytochrome C and release it into the cytoplasm. By using a block copolymer (Pluronic F127) and an organic solvent (1,3,5-trimethylbenzene, TMB) as templates and alkanes/ethanol as pore expanding agents [64], Kao and Mou synthesized a series of pore expanded MSN with the help of inorganic salts (KCl). Among the finite alkanes (hexane, octane, decane, dodecane, and hexadecane), they found decane was the most effective expanding agent, yielding MSN materials with enlarged pores (5.6 nm) and well-ordered mesostructure. They also studied the effects of the amount and ratio of ethanol and ammonia solution (NH_4_OH) on pore size and pore structure. Fan and co-worker demonstrated a new way to construct a large pore size of highly ordered MSN (around 27 nm) with entrance dimensions that varied from 4 to 16.7 nm at a low synthesis temperature [65]. Temperature may be critical to the structures and morphology of this type of MSN due to the flexible shape of the template micelle in low temperature. 

Generally speaking, when MSN size remains fixed, the larger pore size, the bigger volume for protein loading. Kao and Mou synthesized a series of pore-expanded MSNs by using different ratios of decane/ethanol as pore-expanding agents, and then compared the amount of immobilized lysozyme into the pore-expanded MSN and conventional MSN with pore sizes of 5.6 and 2.5 nm, respectively. They found the adsorption capacity of pore-expanded MSN is up to 420 mg lysozyme per gram MSN, which is obviously larger than that of the conventional MSN materials (340 mg lysozyme per gram of MSN) [64], demonstrating the great potential in intra-pore confinement of enzyme and protein.

### 3.2. Surface Functionalization

Silanol groups (Si–OH), presenting on the surface of MSN in the concentration of at least 2–4 groups/nm [66], give the base for decorating MSN with various silyl reagents. MSN can be modified or grafted with different functional groups on its mesopore channel and/or external surface, resulting in various surface groups, zeta potentials, and hydrophilic and hydrophobic properties. The ability to selectively functionalize the external and internal surface of MSNs opens infinite possibilities in the field of drug delivery, especially for protein delivery. The main interactions between the surface groups of MSN and gusts are chemical interaction and physical interaction (such hydrogen bonding and electrostatic interactions). Many studies have demonstrated that the proteins loading capacity of functionalized MSN would be greatly enhanced compared with its un-functionalized or native counterparts [33,34,35]. Owing to negative potential of most proteins, positively charged amino silyl reagents or polymers are the most widely functional groups used for protein adsorption and binding. Table 1 is a summary of complexes of different functional MSN with proteins. Actually, aminosilane functionalized silica nanoparticles have been widely applied to the adsorption and release profile study of proteins, such as lysozyme, bovine serum albumin and myoglobin [37,67]. Ackerman and co-workers reported NH_2_-functionalized mesoporous silica (FMS) with large pore size can realize a highly efficient negatively charged protein confinement or immobilization in comparison with unfunctionalized mesoporous silica and normal porous silica [35]. In the meantime, the loaded negatively charged glucose oxidase (GOX) and glucose isomerase (GI) displayed a comparable immobilization efficiency (I_e_) than the free enzyme in a range from 30% to 160%, 100% to 120%, respectively. Kawi et al. demonstrated that amino functionalized SBA-15 MSNs had more BSA absorption capacity than bare ones due to the strong electrostatic interaction between positively amines and negatively charged BSA [68].

By decorating with citraconic amide functionality on the pore surfaces of mesoporous silica nanoparticle, Lee and co-workers constructed a smart protein delivery system, which could undergo responsive charge conversion in endosomal conditions and release cytochrome c [69]. Lu et al. synthesized a series of functionalized FDU-12 mesoporous silica nanoparticles via co-condensation of etraethoxysilane with a suite of organosilanes, such as 3-aminopropyltriethoxysilane (APTES), 3-mercaptopropyltrimethoxysilane (MPTMS), vinyltrimethoxysilane (VTMS), and phenyltrimethoxysilane (PTMS) by using Pluronic F127 and trimethylbenzene (TMB) as template and pore expanding agent, respectively [36]. They used cellulase, a cellulose-hydrolyzing enzyme as a model protein, and studied the effect of organic functionality on enzyme immobilization efficiency, activity, and stability varied significantly. Due to the size exclusion effects at pore entries from functionality, PTMS and MPTMS functionalized MSN had a very low loading capacity of cellulase. Although APTES functionalized MSN had the highest adsorption efficiency of cellulase by mean of strong electrostatic interactions between amino-functionalized surfaces and enzymes, the enzymatic activity of cellulase would be compromised due to the formation of amide bonds between cellulase and APTES, which led to a loss of active site and change in enzyme spatial structure. Attributed to hydrophobic interaction between cellulase and the vinyl moiety, VTMS-functionalized MSN not only exhibited a strong affinity for the cellulase but also maintained a stable enzyme conformation activity, and appeared to be the most promising approach in these motifs.

### 3.3. Targeting Protein Delivery

Since target tissue/cells have abnormal expression and/or overexpression of particular receptors, the surface of a MSN can be functionalized with corresponding moieties or ligands, such as peptides [70,71,72], proteins [73,74], antibodies [75,76,77] and aptamer [78,79], which have high selectivity and affinity to those receptors. Thus, the functionalized MSN can achieve active targeted delivery of the protein cargoes to its destination. By conjugating TAT cell penetrating peptide and transferrin to the surface of MSNs, Chen and co-workers developed a multifunctional mesoporous silica nanoparticles delivery system for selenoamino acid, a new kind of antitumor drug, achieving synergistic chemo-/radiotherapy through death receptor-mediated extrinsic apoptotic pathway [80]. In this system, TAT peptide and transferrin were employed as targeting ligands to enhance MSN internalization by a cancer cell in vitro and in vivo through receptor-mediated endocytosis.

**Figure 2:**
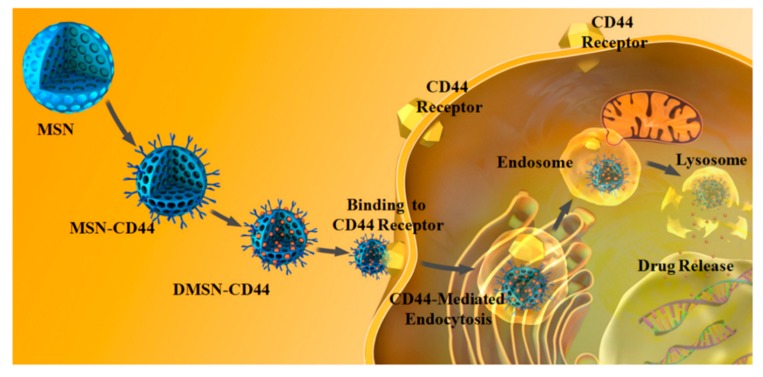
Schematic illustration of an MSN-based protein (CD44 McAb) and chemotherapy drug targeted co-delivery system and overcome multidrug resistance in MCF-7/MDR1 breast cancer cells [83].

It was found that CD44 is highly expressed in some multidrug resistance cancers, such as multidrug resistance (MDR) breast cancer, involving in P-glycoprotein (P-gp) mediated drug efflux and overexpression of the anti-apoptotic protein Bcl-xL [81,82]. Lu and co-workers developed a CD44 monoclonal antibody-functionalized, doxorubicin (DOX)-loaded mesoporous silica nanoparticle as a targeted drug delivery system for enhancing chemosensitivity and overcoming multidrug resistance in vitro and in vivo [83]. CD44 McAb was functionalized on the surface of the MSN through the formation of amide bonds. In the system, CD44 McAb not only served as an active targeting ligand through antigen-antibody reaction but also sensitized the chemotherapeutic efficacy of DOX by reversing the MDR effect (Figure 2).

### 3.4. MSN-Based Combination Therapy of Protein and Other Therapy Models

Many diseases, especially cancer, central nervous diseases, and immunological disease are usually caused by multiple factors. The heterogeneity, complexity of the signaling network and adaptive resistance make them a formidable challenge to defeat. Generally, monotherapy strategy for killing given cells or regulation of a specific target pathway would only yield compromised therapeutic efficacy. Consequently, this can induce the emergence of drug resistance after multiple dosing due to the activation of a compensatory mechanism. Thus, combination therapy with two or more therapeutic agents with complementary or synergistic effect is commonly employed, including the combo of therapeutic proteins and other molecules. In the area of drug delivery, especially nanocarriers, it has been a hot sub-field to develop efficient vehicles that could simultaneously deliver two or more kinds of therapeutic molecules or realize two or more therapeutic models in a coordinated manner. Due to the great difference in physicochemical properties between proteins and other therapeutic molecules, such as size, surface charge, friability, and sensibility, it is a big challenge to accomplish their co-delivery for conventional drug delivery systems. With tunable pore size, various surface functionalization, and enormous interior and exterior particle surface, MSNs could be a promising co-delivery platform for proteins and other therapeutic guest molecules to realize controlled release in a rational manner.

By using hydrophobic indomethacin (IMC) and hydrophilic human peptide YY3-36 (PYY3-36) as model drugs, Santos et al. investigated the potential of MSN for co-delivery drugs with various physicochemical properties, the effect of co-delivery manner on their release profiles and drug-permeation profiles [84]. They revealed that a co-loading procedure could increase the overall loading efficiency of MSN, accelerate drug release rate and promote their permeation behavior. The loading model, single or dual drugs loaded into MSN, had no influence on their permeation profiles. Furthermore, conformational analysis indicated that PYY3-36 could retain its space structures after intestinal cell monolayer permeation and display biological activity after being released from the MSN, indicating promising candidates for the co-loading of hydrophilic and hydrophobic therapeutics.

Shi and co-workers develop chitosan (CS) and mesoporous silica nanoparticle composite hydrogels for the co-delivery of biomacromolecules, bovine serum albumin (BSA), and small chemical drugs gentamicin (GS) [85]. The embedding of MSN in the CS gel would accelerate the gelation rate and enhance its mechanical strength. In addition, MSNs could act as a drugs container and realize cargoes with sustained release. The release of GS and BSA from CS/MSN hydrogels presented a sustained manner simultaneously, compared to single sustained release of BSA from CS hydrogels. In vitro chondrocyte culture demonstrated the CS/MSN composite hydrogels has a great potential in the noninvasive therapy of cartilage regeneration.

**Figure 3:**
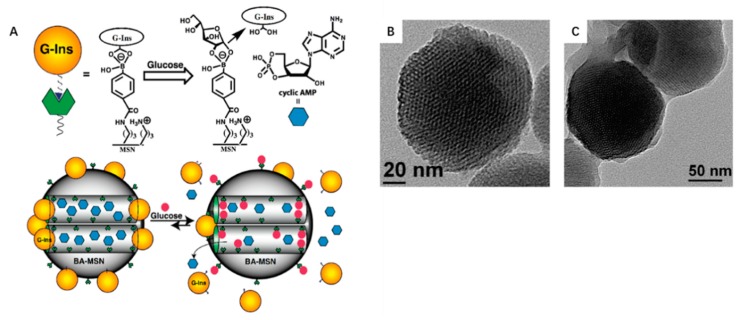
(**A**) Schematic illustration of a double drugs co-delivery system for controlled release of bioactive G-Ins and cAMP. cAMP is confined into the MSN pore; G-Ins is modified on the surface as a pore cap. (**B**) Transmission electron micrograph (TEM) of boronic acid-functionalized MSN. (C)TEM of FITC-G-Ins-capped MSN [39].

Owning to the relative bulk volume and size, proteins drugs can also act as MSN pore blocker to achieve controlled release of drugs. Zhao and co-works synthesized a glucose-responsive MSN based co-delivery system for insulin and cAMP, realizing controlled (Figure 3) sequence release [39]. In the system, insulin modified with gluconic acid and immobilized on the MSN exterior surface through reversible covalent bonding between phenylboronic acid and acyclic diols was not only a model protein drug but also acted as a pore cap to encapsulate cAMP molecules inside the mesopores of MSN. Adjacent diols of saccharides, such as glucose and fructose can competitively form stable cyclic esters with phenylboronic acid than with acyclic diols, thus trigger insulin release from MSN and subsequently the encapsulated cAMP. The cAMP delivery into the cytosol of pancreas beta cells would stimulate insulin secretion. This showed that the triggered insulin release was completed within 30 min, and this interval frame was sufficient for normal insulin secretion. Thus, the decrease of insulin release with repeated cycles of conventional glucose-responsive insulin delivery systems would be overcome by this kind of self-regulated insulin-release [86].

## 4. MSN-Based Stimuli-Responsive Delivery System for Proteins

The stimuli-responsive drug-delivery system (SRDDS) can prevent premature release before reaching the disease foci, and release the encapsulated drugs into targeted locations in response to external stimuli or internal local microenvironment difference, which will increase drug efficacy for targeted cells while decreasing the toxicity to normal cells [87,88]. Due to their unique structure and property, MSNs have attracted a lot attention particularly in SRDDS. Two main methods can achieve responsive drug release from MSN. The first method is to conjugate drugs to the surface of the MSN through cleavable covalent bonds, which can be broken in a specific condition. The other method is to cover the surface of the MSN with a strippable coating or cap the pores of the MSN with blockers as gatekeepers, which would open the pore entrance upon the surrounding environment change, such as pH, redox, enzyme, and temperature, etc.

### 4.1. pH-Responsive Delivery System

pH is the most widely used biological parameter to trigger drug release among various kind of stimuli. Intercellular acidic gradient or distinct difference in pH between the physiological environment and targeting tissue constitute the rationale for pH-responsive drug delivery systems. During the last decade, many pH responsive MSN-based protein delivery systems have been developed.

Due to its acid lability, imine bond formed between primary amine and aldehyde is widely used to construct pH responsive delivery system [89,90]. For example, Han et al. synthesized an aldehyde-functionalized MSN (MSN-aldehyde), which could conveniently and efficiently conjugate with model proteins via imine bonds [91]. To track the protein release in the acidic lysosome, the MSN was labeled with lysosome activatable rhodamine-lactams. They demonstrated that the constructed nanocomposites were selectively internalized into lysosomes of model cells, HepG2, HeLa, and L929 cells. The loaded proteins could be efficiently released from MSN upon the trigger of acidic lysosome solution, followed by escaping into the cytoplasm and exhibiting their corresponding function. Zhu et al. also developed aldehyde-functionalized dendritic mesoporous silica nanoparticles (DMSNs-CHO) with an average particle size of 174 nm and an internal pore size of 7.7 nm as a potential pH-responsive protein drug delivery system [92]. They loaded BSA as a model protein into the pores of DMSNs-CHO and found its release was dependent on environment pH. The DMSNs-CHO nanoparticles could be efficiently taken up by cells and had no cytotoxicity. All those indicate that MSN-aldehydes would be a promising and versatile vector for the delivery of various proteins into cells.

In another example, Lee and coworkers designed and constructed a smart mesoporous silica nanoparticle, whose pore surface was functionalized with pH-hydrolyzable citraconic amides (MSN–Cit), which could undergo charge conversion in acidic lysosome environment [69]. At physiological pH (pH 7.4), MSN-Cit exhibited negative surface charge due to the terminal carboxyl groups of the citraconic amide. Therefore positively charged cytochrome c (Cyt c) could be loaded into the MSN pore through electrostatic interaction. After MSN-Cit was internalized into acidic lysosome (pH 4–5), surface citraconic amide went through hydrolysis, leading to dramatic charge conversion of MSN from negative to positive (16 mV). The charge repulsion between MSN and Cyt c would result in Cyt c release. In vitro release profiles demonstrated only 10% of Cyt c release from MSN-Cit was observed at pH 7.4 in 10 h, whereas more than 30% of Cyt c was release at pH 5.0 for the same period. Confocal laser scanning microscopy (CLSM) studies proved that MSN-Cit effectively released Cyt c in endosomal compartments. Moreover, this charge reversion strategy also endowed MSN-Cit wonderful biocompatibility and the ability for endosomal escaping. Due to the anionic surface nature of red blood cells (RBC), the negative charge at physiological pH 7.4 made the lysis of (RBCs) by MSN-Cit negligible, even at the concentration of 10 mg/mL. In contrast, the charge reversion to positive in acidic endosomes facilitated MSN-Cit absorb on the surface of lysosome membrane and cause the rupture of lysosomes, resulting in the release of Cyt c delivery into the cytoplasm.

### 4.2. Extracellular Glutathione (GSH)-Responsive Delivery System

It is well known that in normal cells, extracellular glutathione (GSH) concentration is approximately 2–10 µM and intracellular concentration is around 2–10 mM [93]. Moreover, it was found that the GSH concentration in cancer cells is several times higher than that in normal cells [94]. This huge difference in GSH concentration between extracellular and intercellular condition makes redox-responsive vehicles a promising carrier to disassemble and release drugs in the cytosol. Utilizing this difference, Griebenow fabricated a redox responsive carbonic anhydrase (CA) controlled release system by immobilizing it onto the internal surface of mesoporous silica nanoparticles via disulfide bond [53]. The internal surface MSN was pre-functionalized with free thiol group by (mercaptopropyl)-trimethoxysilane (MPTMS), and then the model enzyme CA was covalent conjugated to MSN by using sulfosuccinimidyl 6-[3′(2-pyridyldithio)-propionamido]-hexanoate (Sulfo-LC-SPDP) as a linker at the ratio of 1:1. In vitro protein release profiles demonstrated that the system could response to GSH and release CA from MSN. More importantly, the released CA remained at least 80% of its enzyme activity.

**Figure 4:**
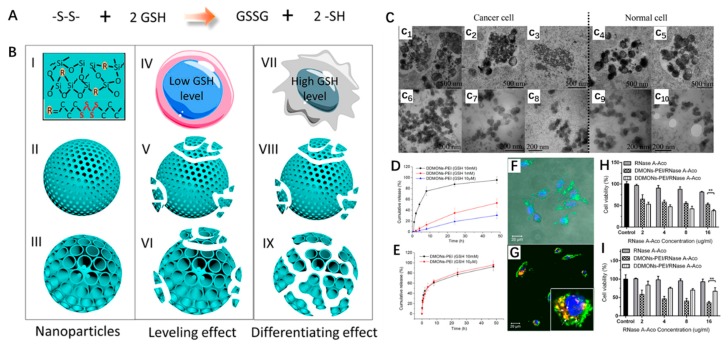
(**A**) The reaction of the disulfide bond is cut off by extracellular glutathione (GSH). (**B**) Schematic illustration of pore structure dependent degradability organic-inorganic hybrid mesoporous silica nanoparticles in normal and cancer cells. (I) organic-inorganic hybrid composition of degradable dendritic mesoporous organosilica nanoparticles (DDMONs), (II, V, VIII) small pore MONs, (III, VI, IX) large pore DDMONs, (IV) normal cell, (VII) cancer cell. (**C**) Intracellular degradation of DDMONs and MONs. DDMONs incubated with B16F10 for 4 h(c1), 24 h(c2), 48 h (c3); DDMONs incubated with HEK293t for 24 h(c2), 48 h(c3); MONs incubated with B16F10 for 4 h(c1), 24 h(c2), 48 h(c3); MONs incubated with HEK293t for 24 h(c2). In vitro release of RNase A-Aco from DDMONs-poly(ethyleneimine)-b-poly (PEI) (**D**) and DMONs-PEI (**E**) in different release solutions. (**F**) The uptake of DDMONs-PEI/RNase A-Aco-FITC complex in in B16F10 cells after 10 h incubation. (**G**) Confocal images of RNase A-Aco-FITC release from DDMONs-PEI in B16F0 cells for 24 h. Cell viability of B16F10 cancer cell (**H**) and Hek293t normal cell (**I**) after incubation with DDMONs−PEI/RNase A-Aco for 48 h (** *p* < 0.01) [95].

Yu et al. first reported a cell-type specific degradable dendritic mesoporous organosilica nanoparticles (DDMONs), which preferentially release protein in cancer cells as opposed to normal cells (Figure 4) [95]. The disulfide bond is homogeneously hybrid into the framework of DDMONs with controllable pore size. The authors studied the pore structure-dependent GSH-responsive degradation behavior in normal cells and tumor cells. They found that only the larger pore DDMONs in tumor cells displayed a much faster degradation rate, indicating efficient protein delivery toward cancer cells, through which selectively kill cancer cells but not normal cells. As a proof of concept, they used ribonuclease A (RNase A) as a model therapeutic protein to demonstrate the benefit of designed DDMONs. In vitro release profiles showed that RNase A displayed obvious GSH-dependent release behavior. It was found that less than 30% of proteins released from DDMONs after 48 h incubation in 10 μM GSH. By contrast, when GSH concentration increased to 1 mM, the release rate of proteins reached ∼57% at 48 h owing to the partial degradation of DDMONs. Surprisingly, when the GSH concentration further increased to 10 mM, a rapid release of protein was observed, reaching ∼97% at 48 h due to the severe rupture of the pore structure. The author further studied the cell inhibition of RNase A loaded DDMONs in cancer cells and normal cells. It found that degradable DDMONs-PEI/RNase A showed higher cytotoxicity than non-degradable DMONs-PEI/RNase A at all concentrations toward B16F10 cancer cells, which is mainly attributed to intracellular high GSH concentration-triggered nanocarriers degradation. In contrast, in normal HEK293t cells, the cytotoxicity of DMONs-PEI/RNase A is significantly lower than that of DDMONs-PEI/RNase A, due to their relatively lower GSH level. All the results demonstrated the disulfide bond hybrid, redox-responsive degradable MSN could be a superior candidate for protein delivery.

### 4.3. Enzyme-Responsive Delivery System

**Figure 5:**
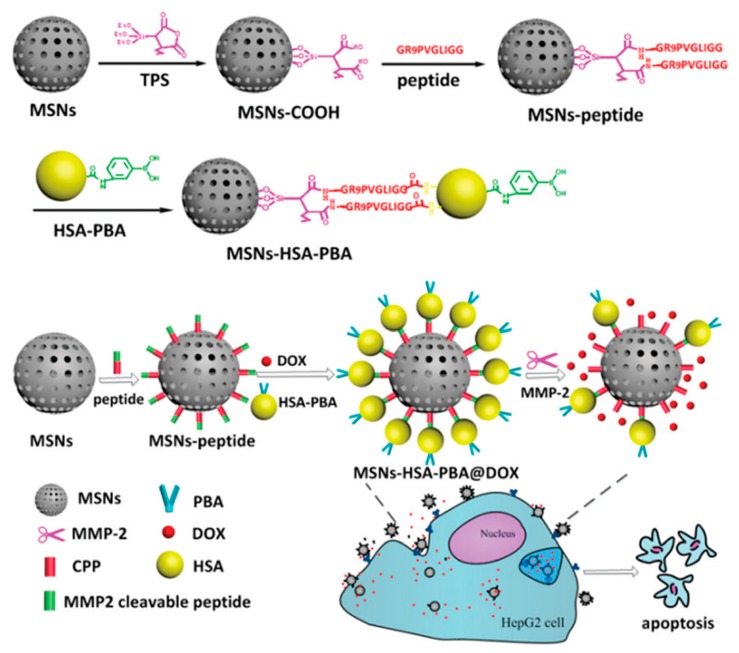
Schematic illustration of the construction of MMP-2 enzyme-responsive mesoporous silica Nanoparticles for the co-delivery of human serum albumin and DOX and its biological effect [99].

Enzymes play a very important role in physiological and pathological processes. The unusual expression or upregulated expression of an enzyme in the target site makes it elegant endogenous stimuli due to its high sensitivity and selectivity [96]. Matrix metalloproteinases-2 (MMP-2) are one of the overexpressed proteins in some tumors, which are closely related to tumor invasion and metastasis [97,98]. Cai et al. employed this trait to construct a MMP-2 responsive BSA and DOX co-delivery system [99]. Phenylboronic acid (PBA) is a highly affinitive ligand for sialic acid (SA), a well-known overexpressed protein indicator for tumor metastasis on HepG2 cells [100]. They decorated PBA to human serum albumin (PBA-HSA) and then used an intermediate linker composed of MMP-2 substrate peptide (PVGLIG) and polyarginine (RRRRRRRRR) to conjugate it onto the surface of MSN, resulting in SNs-HSA-PBA@DOX [99]. Before the linker was cut off by MMP-2, the model protein HSA could not only act as a pore seal to prevent DOX from premature release but also made it convenient to decorate PBA ligand on the surface of the MSN for active targeting. After the breakage of the linker, the opened pore entrance would promote the release of the loaded drugs. Through the integration of active targeting and enzymes’ control drug release strategies, MSNs-HSA-PBA@DOX achieved enhanced antitumor efficacy both in vitro and in vivo (Figure 5).

### 4.4. Heat and Magnetic Responsive Delivery System

Heat is a very effective stimulus which has frequently been used as a trigger for protein release from MSN-based delivery system. To achieve that, such a complex of the MSN system usually includes MSNs, a thermal conversion agent, a drug, and a temperature-sensitive polymer which can swell above certain temperatures (lower critical solution temperature, LCST) and shrink below certain temperatures to facilitate drug release. Stroeve et al. used thermosensitive poly(n-isopropylacrylamide) (PNIPAM) as a gatekeeper to realize controlled bovine hemoglobin (BHb) release [101]. Other than traditional PEGylation on the exterior surface of the MSN to minimize non-specific binding and interactions with the biological environment, they grafted PEG on the interior porous framework to minimize protein adsorption and reduced protein denaturation. Thus, when temperature is raised to higher than the LCST, the PNIPAM gate will open and induce rapidly and sufficiently BHb releasefrom MSN. Vallet-Regí and co-workers showed a novel MSN-based nanocarrier, which could respond to an alternating magnetic field to remotely control the release of loaded small molecules and protein [54]. Iron oxide nanocrystals served as magnetothermal transfer agents were encapsulated inside the MSN matrix. A thermoresponsive copolymer of poly(ethyleneimine)-b-poly(*N*-isopropylacrylamide) (PEI/NIPAM) was decorated onto the surface of MSN and acted as a temperature-responsive gatekeeper for trapping small molecules in the pore matrix as well as a retainer for proteins buried in the polymer shell by electrostatic and hydrogen bonds interactions. When the nanodevices were placed in an oscillating or alternating magnetic field, the iron oxide nanocrystals would transduce magnetic energy to heat due to the hysteresis loss and/or Néel relaxation to promote polarity inversion and tridimensional polymeric network changes if the heat reaches LCST, subsequently provoking the pore opening and a significant molecules and protein release. Moreover, the magnetic nanocrystals would endow the nanodevices’ magnetically targeted ability to the desired place using a permanent magnet. In addition, it can also be employed as contrast agents for magnetic resonance imagining. Similarly, Khashab and co-workers also developed a silica-iron oxide hybrid nanovectors with large mesopores (20–60 nm) for protein (mTFP-Ferritin) loading and delivery to cancer cell [102]. The iron oxide nanophases were homogeneous incorporated into the silica matrix, which made the nanocomplex not only have the ability of magnetically-actuated release of protein but also enhanced the biodegradability of nanovector due to the removal of iron centers from the silica-iron NPs in physiological conditions, exhibiting great promise in biomedical applications.

### 4.5. Light-Responsive Delivery System

Light is an attractive stimulus as a trigger for controlled drug release due to its non-invasive nature, desirable controllability, high spatial resolution and temporal precision with a pulsatile switch on/off model [103,104]. Cargo release profiles from a light-responsive delivery system can be controlled by various light parameters, including wavelength, output power, exposure time, beam diameter, and so on. Liu et al. constructed a light-controlled release, MSN-based protein and model drug co-delivery system [105]. A photo-labile copolymer P(OEGMA-*co*-TENBMA) that could form a protein-polyelectrolyte complex (PPC) with bovine serum albumin (BSA) was anchored on the surface of MSN as a capping agent for the nanopores of the MSNs. The PPC is stable at physiology environment, but P(OEGMA-*co*-TENBMA) trend to hydrolyze to ionic P(OEGMA-*co*-MAA) upon ultraviolet (UV)-irradiation on photo-labile 2-nitrobenzyl ester moieties. This charge conversion would lead to the disruption of PPC and subsequent co-release of BSA and small molecule rhodamine B.

## 5. Biocompatibility

For in vivo biomedical applications, it is crucial that nanoparticles can not only achieve their designed mission but also do not produce non-specific and deleterious changes to the body, indicating good biocompatibility. Although silica has been used as an excipient in the pharmaceutical industry for decades, and generally considered as of low toxicity, its in vivo toxic effects have been reported. Thus its toxicity to the body (including acute toxicity and chronic toxicity) should be carefully assessed when it was applied in the body. The exact mechanism for MSN toxicity is still uncertain, and some theories were proposed and evaluated, including the induction of membrane damage [106], generation of reactive oxygen species [107], disruption of lysosome damage, and interruption of cellular respiration [108]. It has been proven that particle size, shape, structure, and surface function would have a significant influence on MSN biocompatibility. Although a definite conclusion cannot be drawn between MSN physicochemical properties and biocompatibility owing to the complexity of nanotoxicity, the relationship is still controversial, but a relative clarity will pave the way for their application and clinical translation.

**Figure 6:**
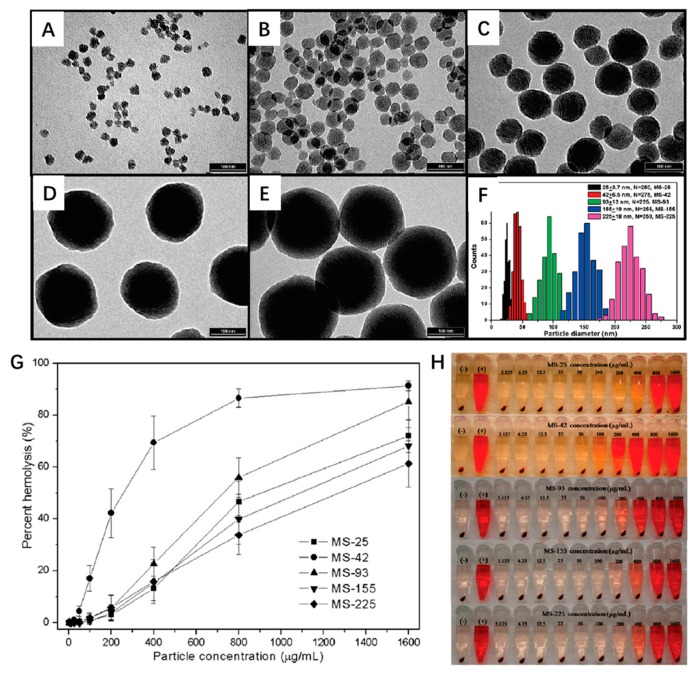
Impacts of mesoporous silica nanoparticle size on hemolytic activity. TEM of mesoporous silica nanoparticles with different size: (**A**) MS-25, 25 nm; (**B**) MS-42, 42 nm; (**C**) MS-93, 93 nm; (**D**) MS-155, 155 nm; (**E**) MS-225, 225 nm; (**F**)The dynamic light scattering size (DLS) distributions of the five mesoporous silica nanoparticles; (**G**) Percentage of hemolysis of red blood cells (RBCs) incubation with different size MS for 3 h in various concentration. (**H**) The photographs of RBCs in G. Water (+) and PBS (−) are used as positive and negative control, respectively [111].

Particle size is one of the most important parameters of MSN, which can have a great influence on MSN biocompatibility. Mou et al. investigated the influence of MSN size on its intracellular internalization by Hela cells [109]. They found that internalized amount of MSN was obvious size-dependent. MSNs with a size of 50 nm showed the highest cellular uptake by HeLa cells, which was around 2.5 times greater than the uptake of 30 nm MSNs; while cellular uptake of 110 nm, 280 nm, and 170 nm MSNs was much lower. The internalized amount of MSNs into Hela cells was in the order 50 nm > 30 nm > 110 nm > 280 nm > 170 nm, and MSN in 50 nm size may be the most effective in drug delivery in the aspect of in vitro cellular uptake. Other research conducted by Li et al. demonstrated that cytotoxicity was highly correlated with the size of MSN [110]. They found all investigated MSNs showed low cytotoxicity at or under the concentration of 25 µg/mL; but when MSN concentration was above 25 µg/mL, MSN with the size of 190 nm and 420 nm showed significant cytotoxicity, while microscale MSN (1220 nm) almost always showed slight cytotoxicity over a broad MSN concentration, ranging from 10 to 480 µg/mL. Lin and Haynes also found that porous MSN would cause a concentration- and size-dependent hemolytic cytotoxicity [111]. The smaller particles exhibited higher toxicity than larger particles (in the range from 25 nm to263 nm) in the concentration range from 3.125 to 1600 µg/mL (Figure 6).

Structural properties of MSN could also affect its biocompatibility, and this effect is especially prominent when it compares to amorphous silica in its hemolytic property. RBCs would be lysed when amorphous silica is at a high concentration. The mechanism accounts for this hemolysis cytotoxicity is mainly based on the interaction between the numerous surface silanol groups of silica materials and the tetra-alkyl ammonium groups on the membrane of RBCs. Although compared with amorphous silica material, highly ordered MSN had more silanols due to a large surface area, but most silanol groups were on the internal surface, which could be attributed to the less negative ζ-potential of the highly ordered MSN (−35 mV) than the one of amorphous silica (−49 mV). The relatively low concentration of external silanols made MSN no-hemolytic on RBCs under determined concentration. Furthermore, the discontinuous external surface silanols due to mesoporosity would lower the hemolytic activity of MSN [112]. Similarly, the shape of MSNs also has some effect on their biocompatibility. Yu et al. constructed several kinds of MSNs with different shapes and studied shape effect on cytotoxicity [113]. Although they found that aspect ratio, ratio of length over width, of MSN had no significant impact on its acute cytotoxicity, cellular uptake, proliferation inhibition, and plasma membrane integrity in given cell lines, they had a great influence on its hemolytic activity. The higher the aspect ratio is, the lower the hemolytic toxicity. This effect mainly contributed to differential silanol density of each nanoparticle. Larger aspect ratio means smaller surface area, logically less silanol exposed to RBCs, which subsequently yields little hemolytic activity. Besides the aspect ratio, the shape of MSN also has a significant influence on cellular uptake in vitro. Lin et al. demonstrated the effect of MSN shape (spherical- and tube-like morphology) on the endocytosis in different cell lines [114]. They found that this effect is morphology and cell line dependent. Chinese hamster ovarian (CHO) cells had a rapid and similar endocytosis speed and rate for sphere MSNs (80 to 150 nm) and tube-shaped MSNs (aspect ratio of 6), while the endocytic capability of fibroblast cells for sphere MSNs was significantly higher than that for rod-like MSNs.

The surface property or surface functionalization is another crucial parameter apart from size, which not only has significant influence on cargoes’ delivery but is also involved in MSN vehicle biocompatibility. As mentioned above, abundant silanol groups exposed on a bared MSN surface provide facile rivet for surface modification. Different modifications could interact with cellular membrane lipids and proteins and disturb their structure and conformation. Positive charge reagents or polymers are commonly used in protein delivery MSN systems. It is well known that particles with cationic surface charge would induce intense immune response, cytotoxicity, and short circulation times compared with anionic and neutral ones. However, positive surface charge is of obvious superiority for transvascular transport and intracellular transport due to the generally negative potential of the cell membrane [115]. Thus, how to finely tune the surface modification for MSN is pivotal in improving MSN biocompatibility and efficacy. PEGylation is the most popular and efficient modification approach to improve nanoparticles’ biocompatibility. It has been demonstrated that polyethylene glycols (PEGs) decorated on nanoparticles can form a hydrophilic corona around the particles, which will increase particle dispersity, decrease the endocytosis and increase circulation time in vivo by delaying opsonization [116,117]. After being modified with PEG, it is easy to imagine that the surface silanol groups on the MSNs would be masked by a PEG layer. Many studies have given plenty of robust evidence that the PEGylation in MSN could ameliorate the hemolytic cytotoxicity, decrease its endocytosis and distribution in mononuclear phagocytic system organs of the liver and spleen, and prolong its half-life in blood [118,119,120,121,122,123]. However, the induction of specific anti-PEG IgM after repeated injection of PEGylated particles would accelerate the blood clearance of PEGylated nanoparticles counterproductively [124] and also give rise to a hypersensitivity reaction led by complementary cascade activation [125]. Other surface functional groups have also been applied to improve MSN biocompatibility, manipulate their in vivo distribution and excretion, such as amino (–NH2), carboxyl (–COOH), phenyl (–Ph) and methyl phosphonate [32,126,127,128], even including some lipid layer [129,130,131,132,133] and bio-membranes derived from various cells [134,135,136].

## 6. Conclusions and Outlook

In this review, we mainly focus on research advances on MSN-based protein delivery. It is clear that MSN materials are promising candidate carriers for protein delivery in vitro and in vivo due to their specific structure and physicochemical property. First, their versatile and adjustable pore size and porosity make MSNs suitable for loading proteins cargos. Second, the simple surface functionality broadens their protein delivery ability and possibility, such as different kinds of proteins, co-delivery of proteins and other drugs, targeted protein transport, and various stimuli-responsive protein release methods. Third, their great biocompatibility bestows promising potential for future biomedical applications.

Besides the aforementioned advantages, there remain some significant challenges that should be investigated to enable the development of practical applications for MSN-based protein delivery systems. Apart from in vitro protein release profiles’ investigation, much more attention should be paid to track in vivo navigation of proteins, especially their release in target tissues by using integrated multifunctional or multimode imaging methods. Although there has been some research into the development of such multifunctional MSNs, novel-innovative methods for functionalization remain limited. Protein structure is the base of its function. In view of the frangibility of proteins and complexity in a physiological and pathological conditions, there should be caution characterizing their structures, as well as the biofunction after their release from MSN in the targeted site. Toxicity is the major obstacle for the translation of nanomaterials from preclinical research to clinical application. Although most recent reports showed that MSN materials have little cytotoxicity, and are of great biocompatibility in vivo, much more work still needs to be performed to demonstrate comprehensively the biological safety of MSNs, such as the ultimate fates of MSNs after in vivo application.

## Figures and Tables

**Table 1:** Functional mesoporous silica nanoparticles for protein delivery.

The Type of MSN	Modification	Proteins	Cell or Disease Models	References
MSN	Propylthiol	Cytochrome C	HeLa cells	[34]
MSN	Amino (–NH2), carboxyl (–COOH)	Glucose oxidase (GOX) and glucose isomerase (GI)	No	[35]
FDU-12	Aminopropyltriethoxysilane (APTES), 3-mercaptopropyltrimethoxysilane (MPTMS), vinyltrimethoxysilane (VTMS), and phenyltrimethoxysilane (PTMS)	Bovine serum albumin	No	[36]
BSA-15	Aminosilanes	Lysozyme (LYS) and Myoglobin (MYO)	No	[37]
BSA-15	Aminosilanes	Bovine serum albumin (BSA)	No	[38]
MSN	Boronic acid	Insulin and cyclic adenosine monophosphate (cAMP)	Rat pancreatic RIN-5F cells	[39]
MSN	Glycidoxypropyltrimethoxysilane (GPTMS), chitosan	Bone morphogenetic protein-2 (BMP-2)	Bone mesenchymal stem cells (bMSCs), Bone regeneration	[40]
MSN	3-aminopropyltrimethoxysilane (APTMS)	Superoxide dismutase (SOD) and glutathione peroxidase (GPx)	Inflammation and oxidative stress	[41]
MSN	3-aminopropyltrimethoxysilane (APTMS)	Superoxide dismutase (SOD)	HeLa cells	[42]
BSA-15	3-aminopropyltriethoxysilane (APTMS)	Bone morphogenetic protein 2 (BMP-2)	Bone marrow stromal cells (BMSCs)	[43]
MSN	2-[methoxy(polyethylenoxy)-propyl] trimethoxysilane (PEG-silane)	Luciferase	Hela cells	[44]
Hollow mesoporous silica capsules (HMSCs)	Carboxyl, Amino, 5-aminofuorescein (AFL)	BSA, Goat IgG	HeLa cells	[45]
MSN	2-(methoxy [polyethyleneoxy]propyl) trimethoxysilane	BSA, macrophage colony-stimulating factor and receptor	Zebrafish embryos/larvae	[46]
MSN	Soluble CD4 (“sCD4”), amide-immobilized sCD4, 18-peptide CD4 fragment	HIV-1 gp120 Glycoprotein	No	[47]
SBA-15	Unmodified	Porcine pancreas lipase (PPL)	Catalyst	[48]
MSN	Unmodified	β-galactosidase	N2a cell	[49]
MSN	Gold nanoparticle	BSA and enhanced green fluorescent protein (eGFP)	Onion epidermis cells (plant cell)	[50]
MSN	PEGylated	BSA	No	[51]
SBA-15	3-aminopropyltriethoxysilane (APTES), 3-mercaptopropyltrimethoxysilane (MPTMS), phenyltrimethoxysilane (PTMS), vinyltriethoxysilane (VTES), and 4-(triethoxysilyl)butyronitrile (TSBN)	Penicillin G acylase (PGA)	No	[52]
MSN	3-aminopropyltriethoxysilane (APTES)	Carbonic anhydrase (CA)	Human cervical cancer (HeLa) cells	[53]
MSN	Poly(ethyleneimine)-b-poly(*N*-isopropylacrylamide) (PEI/NIPAM)	Trypsin inhibitor protein (type II−S), catalase	No	[54]
